# Occurrence and Distribution of *Apolygus lucorum* on Weed Hosts and Tea Plants in Tea Plantation Ecosystems

**DOI:** 10.3390/insects10060167

**Published:** 2019-06-11

**Authors:** Yueyue Tian, Hanyue Wang, Jian Hou, Lixia Zhang, Zhengqun Zhang, Xiaoming Cai

**Affiliations:** 1 College of Horticulture Science and Engineering, Shandong Agricultural University, Tai’an 271018, China; yueyueer8023@163.com (Y.T.); chaxuewanghanyue@163.com (H.W.); zlx_sdau@163.com (L.Z.); 2 Lanshan Tea Administration, Rizhao 276808, China; houjiantaian@163.com; 3 Tea Research Institute, Chinese Academy of Agricultural Science, Hangzhou 310008, China

**Keywords:** *Apolygus lucorum*, tea plants, weed hosts, population change

## Abstract

The mirid bugs are one of the most important piercing–sucking insect pests in tea plantations, which severely reduce the quality and economic benefits of tea. In this study, the mirid bug species in the three tea-producing areas in Shandong Province of China were investigated. The distribution and occurrence of dominant species of mirid bugs on four weed host plants and tea plants *Camellia sinensis* (L.) O. Kuntze (Theaceae) were also studied in the tea agro-ecosystems. The results showed that *Apolygus lucorum* (Meyer-Dür) (Hemiptera: Miridae) was the dominant mirid bug species in the tea growing areas. *Apolygus lucorum* densities on *Humulus scandens* (Lour.) (Moraceae) and *Artemisia lavandulaefolia* DC. (Asteraceae) were relatively higher than those on *Conyza canadensis* (Linn) Cronq (Asteraceae), *Artemisia annua* Linn (Asteraceae), and *C. sinensis*. Host plant switching of *A. lucorum* in the tea agro-ecosystem was: *A. lucorum* scattered on and seriously infested tea plants in June and July; *A. lucorum* largely migrated to and gathered on *H. scandens*, *A. lavandulaefolia*, *C. canadensis*, and *A. annua* at the flowering stage, and population densities of *A. lucorum* on these flowering hosts peaked in late September; in October, *A. lucorum* gradually moved back to flowering tea plants. These results could provide a reference for selecting host plants, such as *Artemisia* plants, as trap plants for sustainable control of mirid bugs in tea plantations.

## 1. Introduction

**Figure 1:**
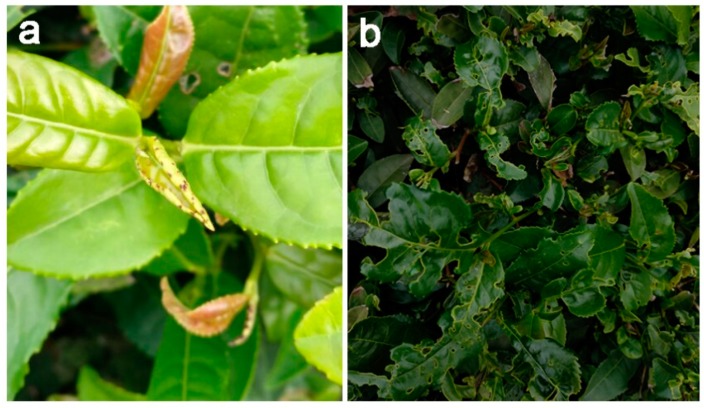
Young tea shoots (**a**) and tea leaves (**b**) damaged from probing by mirid bugs.

Mirid bugs (Hemiptera: Miridae) are omnivorous species in natural and agricultural ecosystems throughout the world, and exhibit diverse feeding habits (e.g., feeding on leaf, stem, inflorescences, fruit, and small insects) [1,2]. These pestiferous insects can seriously damage a variety of agricultural crops, including cotton, legumes, cereals, vegetables, fruits, and tea plants [3]. In recent years, mirid bugs have become one of the most common and economically important piercing–sucking herbivores of tea plants *Camellia sinensis* (L.) O. Kuntze (Theaceae). Damage of these insect pests to tea plants has been increasing and harmful in some tea growing regions of northern China [4]. Both adults and nymphs suck the sap from young buds and shoots of tea plants. Small reddish-brown dead spots on the younger tea buds are caused by stylet probing just after infestation of mirid bugs (Figure 1a). The spots of dead tissue gradually form irregular holes in the leaves, or cause the tea leaves to become fragmentary as the buds grow (Figure 1b) [3]. The digestive enzyme polygalacturonase in the salivary glands of mirid bugs plays the most important role in the induction of visible injury on the leaves of tea plants [5]. Damage to tea plants caused by feeding of mirid bugs results in a loss of 30–40% of tender tea shoots during the spring growing season depending on the pest population density, and causes large economic losses and reduction in tea quality [4].

Mirid bugs have a broad range of host plants, and adults have the capability to undertake long-distance flight among different host plants [6]. For example, 288 plant species in 54 families were found to be the hosts of *Apolygus lucorum* (Meyer-Dür) (Hemiptera: Miridae) [7,8]. Host switching in *A. lucorum* might be likely determined by seasonal population density of this insect and host plant availability [9]. In particular, *A. lucorum* adults switched host plants according to the succession of flowering plant species in the local agro-ecosystem [9]. *Humulus scandens* (Lour.) (Moraceae), *Artemisia lavandulaefolia* DC. (Asteraceae), *Conyza canadensis* (Linn) Cronq (Asteraceae), and *Artemisia annua* Linn (Asteraceae) are the common weed species which are easy to grow in the agro-ecosystem and have a long blooming period in autumn. Pan et al. [9] found that *A. lucorum* adults preferred these weed plant species in bloom. However, very little is known about the distribution and host plant switching of mirid bugs in the tea growing areas of northern China, where these insect pests occur seriously. 

In the present study, field trials were carried out to investigate the distribution and occurrence of mirid bugs on the tea plants and the four weed plant hosts in the tea agro-ecosystem in Shandong Province of China in 2016 and 2018. Seasonal population change of the dominant mirid bug species was also monitored in 2016. The data can be used to understand the interaction between mirid bugs and host plants and further select specific weed plant hosts as trap plants in area-wide management tactics for mirid bugs in the Chinese tea plantations.

## 2. Materials and Methods

### 2.1. Field Experimental Design 

The experiments were conducted in three commercial tea plantations in Tai’an (experiment site 1: 36.221° N, 116.943° E; experiment site 2: 36.120° N, 117.145° E), Rizhao (35.207° N, 119.246° E), and Qingdao (36.266° N, 120.651° E), Shandong Province, China. *H. scandens*, *A. lavandulaefolia*, *C. canadensis*, and *A. annua* are the common and important weed plant hosts of polyphagous mirid bugs in the agro-ecosystem [1,9]. Four wild weed plant species as potential trap plants were transplanted as seedlings from nearby agricultural fields to the tea plantations. Each weed plant species was established in four separate 4 × 2 m plots in early May in both 2016 and 2018, respectively. The seedlings of each weed plant species were planted in nine rows with distances of 25 cm between the rows and 25 cm between plants within the rows. The individual plots were positioned at random and spaced at 3 m intervals. The area planted with potential trap plants was approximately 500 m^2^, and embedded within the >10 ha tea plantations. 

### 2.2. Plot Surveys

The distribution of mirid bugs in three tea-producing areas and the numbers of the dominant mirid bug species on *C. sinensis*, *H. scandens*, *A. lavandulaefolia*, *C. canadensis*, and *A. annua* in tea plantation were investigated on 20 September 2016 and on 20 September 2018. The number of mirid bugs in each plot was determined by the knock-down method complemented by visually inspecting plants following Zhang et al. [4] and Pan et al. [9]. Both sampling methods were directed to the upper parts of plants. Knock-down techniques consisted of pulling parts of the tea plants over a rectangular white-colored pan (60 × 35 × 3 cm), after which the plant material was struck five times and the number of dislodged bugs was counted. During the sampling process, the number of mirid bugs was determined by both sampling methods. The mirid bug species, sex and age of individuals were subsequently identified based upon morphological features according to Jiang et al. [3]. Subplots of 1 × 1 m were sampled within each plot. To monitor the population change of the dominant mirid bug species *A. lucorum* on different host plants, sampling was conducted at 10 day intervals in each plot from 5 June to 15 October 2016 in the tea plantations in Daiyue county (36.221° N, 116.943° E) (experiment site 1) and Taishan county (36.120° N, 117.145° E) (experiment site 2) in Tai’an, Shandong Province, China.

### 2.3. Statistical Analysis

All statistical analyses were performed using SPSS statistical software (version 18.0, SPSS Inc., Chicago, IL, USA). Generalized linear models (GLM) were used in order to know the effects of mirid bug species, tea-producing area and interactions on the numbers of mirid bugs, and the effects of host plant species, tea-producing area and interactions on the number of *A. lucorum*, respectively. Statistically significant mean values of the numbers of mirid bugs were determined by Tukey’s HSD method (*p* < 0.05). GLM followed by Tukey’s HSD tests (*p* < 0.05) were also used to compare numbers of females, males, and nymphs of *A. lucorum* on four different host plants. Significant differences in the *A. lucorum* population density on the four weed plant hosts and tea plants were determined using repeated-measures analysis of variance (MANOVA), with host plants as the factors and the sample date as the split-plot factor. Multiple comparison in the abundance of *A. lucorum* among different host plant species was also determined through Tukey’s HSD tests (*p* < 0.05).

## 3. Results

### 3.1. Dominant Mirid Bug Species in Tea Agro-Ecosystems

**Figure 2:**
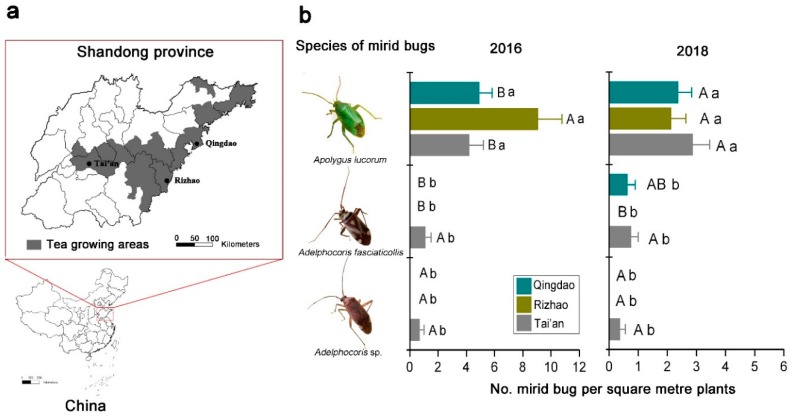
Tea growing areas in Shandong province of China (**a**) and the distribution of mirid bugs in three tea-producing areas in Shandong Province in 2016 and 2018 (**b**). Different uppercase and lowercase letters indicate significant differences among different tea-producing areas and different mirid bug species, respectively (Tukey’s HSD test, *p* < 0.05).

There were significant differences in mirid bug species in the tea agro-ecosystem among the three different tea growing areas (in 2016: *F* = 45.267, *p* < 0.001; in 2018: *F* = 34.225, *p* < 0.001). The interaction between mirid bug species and tea-producing area also significantly affected the numbers of mirid bugs (in 2016: *F* = 11.735, *p* < 0.001; in 2018: *F* = 41.279, *p* < 0.001) (Table 1). *Apolygus lucorum* was the dominant species in the tea growing areas in Shandong Province of China. In 2016, the population density of *A. lucorum* in the tea agro-ecosystem in Rizhao was 9.07 ± 1.70 per square meter plants, and it was significantly higher than in Qingdao (4.93 ± 0.89 per square meter plants) and Tai’an (4.20 ± 0.10 per square meter plants) (*F*_2,29_ = 12.412, *p* < 0.001). In addition, little *Adelphocoris fasciaticollis* Reuter (1.10 ± 0.41 per square meter plants) and *Adelphocoris* sp. (0.70 ± 0.30 per square meter plants) were found in Tai’an. In 2018, there was no significant difference in the *A. lucorum* density in all sampling sites (Qingdao: 2.38 ± 0.46 per square meter plants; Rizhao: 2.13 ± 0.52 per square meter plants; Tai’an: 2.88 ± 0.58 per square meter plants) (*F*_2,29_ = 0.537, *p* = 0.592). For *A. fasciaticollis*, the density of this mirid bug species in Tai’an was significantly higher than in Rizhao in 2018 (*F*_2,29_ = 3.678, *p* = 0.043) (Figure 2).

### 3.2. Distribution of *A. lucorum* on Main Hosts in Tea Agro-Ecosystems

**Figure 3:**
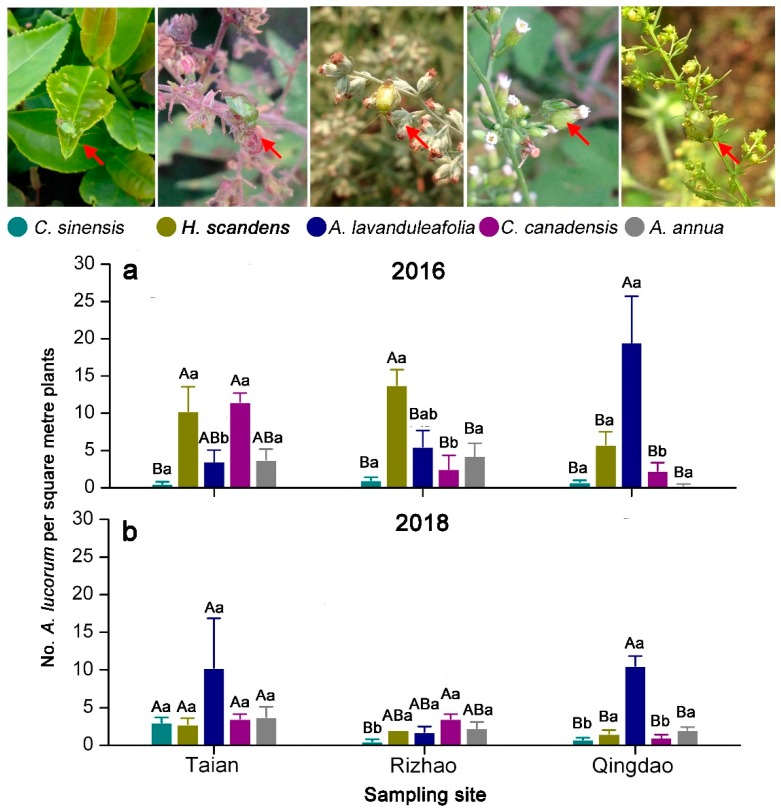
Number of *Apolygus lucorum* on *Camellia sinensis*, *Humulus scandens*, *Artemisia lavandulaefolia*, *Conyza canadensis*, and *Artemisia annua* in tea plantation in different tea-producing areas in 2016 (**a**) and 2018 (**b**). Different uppercase and lowercase letters indicate significant differences among different tea-producing areas and different mirid bug species, respectively (Tukey’s HSD test, *p* < 0.05). The red arrows indicate *A. lucorum* on tea plants and four weed host plants at flowering stage.

There was significant difference in *A. lucorum* density among different host plant species (in 2016: *F* = 5.485, *p* < 0.001; in 2018: *F* = 4.749, *p* = 0.002). However, the tea-producing area did not significantly affect the numbers of *A. lucorum* (in 2016: *F* = 0.035, *p* < 0.965; in 2018: *F* = 2.383, *p* = 0.102). The interaction between host plant species and tea-producing area had significant effect on numbers of *A. lucorum* in 2016 (*F* = 6.190, *p* < 0.001) (Table 2). In 2016, the *A. lucorum* densities on *C. canadensis* (11.50 ± 1.19 per square meter plants) and *H. scandens* (10.25 ± 3.30 per square meter plants) were significantly higher than on *C. sinensis*, but not significantly different from *A. lavandulaefolia* and *A. annua* in the tea agro-ecosystem in Tai’an (*F*_2,23_ = 6.654, *p* = 0.003). In Rizhao, the *A. lucorum* density on *H. scandens* was 13.75 per square meter plants, and it was significantly higher than on tea plants and other two weed host plants (*F*_2,23_ = 7.906, *p* = 0.001). In Qingdao, among the different host plants, the highest *A. lucorum* density was found on *A. lavandulaefolia* (*F*_2,23_ = 7.487, *p* = 0.002). The *A. lucorum* density on *C. canadensis* in Tai’an was significantly higher than in Rizhao and Qingdao (*F*_2,11_ = 17.143, *p* < 0.001). In Qingdao, on *A. lavandulaefolia*, *A. lucorum* exhibited higher population density than in Tai’an and Rizhao (*F*_2,11_ = 5.002, *p* = 0.035). The *A. lucorum* densities on the tea plants, *H. scandens,* and *A. annua* were not significantly different among the three different sampling sites, respectively (Figure 3a). In 2018, the *A. lucorum* density on tea plants in Tai’an was significantly higher than in Rizhao and Qingdao (*F*_2,11_ = 8.806, *p* = 0.008). *Apolygus lucorum* populations were not significantly different across host plant species in Tai’an (*F*_2,23_ = 1.057, *p* = 0.411). The density of *A. lucorum* was significantly higher on *C. canadensis* compared with *C. sinensis* in Rizhao (*F*_2,23_ = 3.227, *p* = 0.042). In Qingdao, more *A. lucorum* were found on *A. lavandulaefolia* compared with the other host plant species (*F*_2,23_ = 35.713, *p* < 0.001) (Figure 3b).

**Figure 4:**
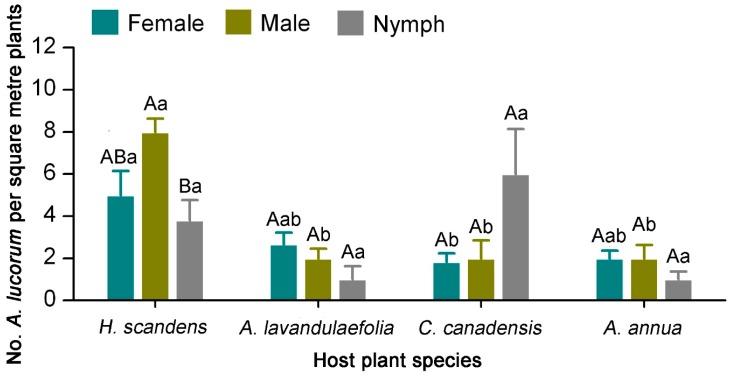
Number of females, males, and nymphs of *A. lucorum* on four different host plants in tea plantation. Different uppercase letters indicate significant differences among males, females and nymphs and different lowercase letters indicate significant differences among different host plants (Tukey’s HSD test, *p* < 0.05).

In early September, the number of male *A. lucorum* adults on *H. scandens* was not significantly different compared with female adults, but was significantly higher than nymphs (*F*_2,11_ = 5.318, *p* = 0.022). There were no significant differences in the number of males, females, and nymphs of *A. lucorum* on *A. lavandulaefolia*, *C. canadensis*, and *A. annua*, respectively. The number of female *A. lucorum* adults on *H. scandens* was significantly higher than on *C. canadensis*, but was not significantly different compared with *A. lavandulaefolia* and *A. annua* (*F*_3,19_ = 4.001, *p* = 0.027). The number of male *A. lucorum* adults on *H. scandens* was significantly higher than on the other three host plants (*F*_3,19_ = 17.143, *p* < 0.001). There was no significant difference in the number of *A. lucorum* nymphs among the four host plants (*F*_3,19_ = 2.919, *p* = 0.066) (Figure 4).

### 3.3. Population Change of *A. lucorum* on Tea Plants and Weed Hosts

**Figure 5:**
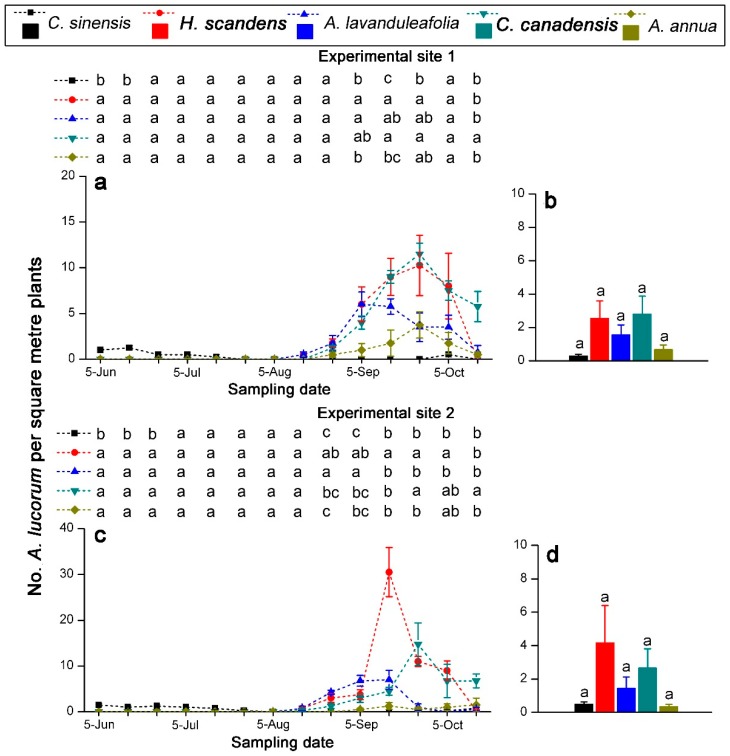
Seasonal changes of population density (**a**,**c**) and seasonal density (**b**,**d**) of *A. lucorum* on tea plants and four weed host plants in the tea agro-ecosystem in 2016. Different letters indicate significant differences among different host plants (Tukey’s HSD test, *p* < 0.05).

In experimental site 1, *A. lucorum* infested tea plants in the tea agro-ecosystem in June and July. The highest population density of *A. lucorum* occurred on the tea plants on 15 June 2016, reaching 1.25 ± 0.25 per square meter plants. In mid-August, the host plants of *A. lucorum* such as *H. scandens*, *A. lavandulaefolia*, *C. canadensis*, and *A. annua* began to flower and bloom. *Apolygus lucorum* largely migrated to these flowering plants and gathered on them. On 5 September, *A. lucorum* density on *A. lavandulaefolia* reached its maximum value of 6.00 ± 1.35 per square meter plants. *Apolygus lucorum* reached peak abundance on *H. scandens*, *C. canadensis*, and *A. annua* on 25 September (*H. scandens*: 10.25 ± 3.30 per square meter plants; *C. canadensis*: 11.50 ± 1.19 per square meter plants; *A. annua*: 3.75 ± 1.44 per square meter plants). In October, the number of *A. lucorum* on *H. scandens*, *A. lavandulaefolia*, *C. canadensis*, and *A. annua* declined gradually, and these insects were found on the tea plants again (Figure 5a). In experimental site 2, *A. lucorum* was also found on tea plants in June and July. The highest population density of *A. lucorum* on *H. scandens* occurred in mid-September, and was significantly higher than the densities on tea plants and other three weed hosts (*F*_4,19_ = 23.235, *p* < 0.001). The population density of *A. lucorum* on *A. lavandulaefolia* peaked on 25 September. However, this value was significantly higher than *C. canadensis*, *A. annua*, and tea plants, but not significantly different compared with *H. scandens* (*F*_4,19_ = 9.972, *p* < 0.001). In October, *A. lucorum* migrated into the tea plantations (Figure 5c). Overall, seasonal density of *A. lucorum* on tea plants was not significantly different compared with densities on four weed hosts as potential trap plants in two of the experimental sites (Figure 5b,d).

The host plants and sampling date significantly affected the number of *A. lucorum* on the host plants in the tea agro-ecosystem (*p* < 0.001). Furthermore, the interaction between host plants and sampling date also had a significant effect on population change of *A. lucorum* (*p* < 0.001) (Table 3).

## 4. Discussion

*Apolygus lucorum* was the dominant mirid bug species on *C. sinensis* in the tea growing areas in Shandong Province of China. Meanwhile, a small amount of *Adelphocoris* spp. occurred in some tea gardens (Figure 2). *Apolygus lucorum* and *Adelphocoris* spp. can infest a wide variety of crops including cotton, Chinese dates, apples, pears, grapes, and tea plants in northern China, and the mirid bugs cause serious yield losses [10,11]. For example, *A. lucorum* and *Adelphocoris* spp. have emerged as economically important insect pests of cotton for thirty years and cause serious yield losses in the Yangtze River and the Yellow River cotton growing regions of China [12,13,14]. It is noteworthy that *A. lucorum* is the dominant mirid bug species in Chinese agro-landscapes and has become the primary pest of several key agricultural crops over the past decade [11].

In the tea plantations, *A. lucorum* adults laid eggs inside some tea plant tissues, such as the pith of dead branches with pruning wounds and the floral receptacle [15]. In April, the overwintering eggs of *A. lucorum* begin to hatch and the newly emerged nymphs feed on tender buds of tea plants. During the initial period of infestation, the highly mobile mirid bug nymphs can induce significant cryptic damage which is difficult to be detected [16]. Therefore, mirid bugs, mainly *A. lucorum*, can seriously damage tea plants in early spring, and cause estimated tea yield losses ranging from 30% to 40% of the total tea tender shoots [4]. *Apolygus lucorum* adults also prefer to deposit eggs for overwintering in the dead parts of branches of fruit trees such as the Chinese date *Ziziphus jujuba* Mill. and the grape *Vitis vinifera* L. where the eggs are less likely to be affected by plant growth, which makes weather conditions the most important factor evoking egg hatching [7,16]. Overwintering eggs of *A. lucorum* begin to hatch in April, and the newly emerged nymphs feed on the tender leaves, buds and flowers and cause serious damage. Weather conditions, especially rainfall, were the most important factor evoking overwintering eggs of *A. lucorum* hatching in the dead parts of tree hosts [3,16]. Therefore, during the spring season, rainfall can strongly affect the population densities of *A. lucorum* in agroecological systems which determine the degree of plant damage.

The polyphagous species *A. lucorum* prefers to feed on the relatively energy-rich plant tissues in flowers and buds. To locate suitable food, *A. lucorum* adults showed a clear preference for flowering host plants and switched food plants according to the succession of different flowering plant species [1,9]. In late August, *A. lucorum* gathered on *H. scandens* and Artemisia plants at flowering stage in the tea agro-ecosystem (Figure 5). The high preferences of *A. lucorum* on flowering plants indicated that flowers and flower nectars might be an optimal food for this pest, and some nutrients from flowers might be the most important for its survival, development, and fecundity [17]. This strategy of host plant switching also can avoid intra- and interspecific competition of mirid bugs for host plants [9]. The preferences of *A. lucorum* on flowering hosts were not just for feeding, but also for female oviposition [18]. Moreover, *A. lucorum* adults and nymphs usually have similar feeding habits, with both life stages consuming plant tissues, nectar, and pollen [17,19]. Both adults and nymphs simultaneously existed on the flowering hosts indicated that some eggs of *A. lucorum* were laid and hatched and the fifth generation was completed on these host plants (Figure 4). In October, population density of *A. lucorum* on *H. scandens*, *A. lavandulaefolia*, *C. canadensis* and *A. annua* declined gradually at the end of the bloom period, and this pest was detected on the tea plants which are at full-bloom stage (Figure 5). Dong et al. [17] found that female *A. lucorum* preferred to lay significantly more eggs on flowering host plants. After supplementing with nutrients on the flowering weed hosts, *A. lucorum* adults of the fifth generation moved back into tea gardens to lay eggs in the dead parts of tea plants for overwintering. *Apolygus lucorum* density in the tea plantations might be positively associated with the abundance of flowering of tea plants. Further investigation would be required to demonstrate the movement of *A. lucorum* adults from weed hosts to tea fields at the landscape level exploiting the plant DNA detection approach [18,20]. In conclusion, our elucidation of occurrence and distribution of *A. lucorum* on weed hosts and tea plants will contribute to the development of sustainable management strategies, such as “push–pull” strategy, for *A. lucorum* in the tea plantation ecosystem.

The “push–pull” habitat management strategy, as a new powerful and effective tool in integrated pest management, uses a combination of behavior-modifying stimuli to manipulate the distribution and abundance of pests and/or their natural enemies for pest control [21]. In this system, highly attractant trap plants release behavior-manipulating semiochemicals that attract the targeted pests to expected areas in which they are subsequently concentrated. One of the most well-known systems is the “push–pull” strategy for the control of cereal stemborers in maize-based farming system in eastern Africa [21]. Napier grass *Pennisetum purpureum* and Sudan grass *Sorghum sudanense* releasing six active compounds effectively attract stemborer moths from maize crop [22,23]. Previous study showed that *Vigna radiatus* had been identified as a potential trap crop for *A. lucorum* in Bt cotton fields [24]. During the autumn season, *A. lucorum* largely aggregated on flowering weed hosts which were chosen as potential trap plants in the tea agro-ecosystem. The *A. lucorum* densities on *H. scandens* and *A. lavandulaefolia* were significantly higher than on tea plants when these weed hosts were at the flowering stage in September and October (Figure 3 and Figure 5). Active compounds such as butyl acrylate, butyl propionate, and butyl butyrate released by *H. scandens* and *Artemisia* plants at the flowering stage mediated *A. lucorum*’s preference to flowering host plants [25]. Hence, the flowering host plants planted in tea plantations can be used as trap plants to attract and aggregate *A. lucorum* due to host plant–mired bug interactions and their emission of behavior-manipulating semiochemicals. Then, some chemical or physical measures could be used to eliminate *A. lucorum* gathering on these plants to avoid these pests migrating on tea plants again. Because *H. scandens* and *C. canadensis* are the vicious weed species in tea gardens, the prospect of using these plants as trap plants for manage *A. lucorum* is negligible. *Artemisia* plants are nontoxic and safe to organisms and the environment and can also be used in traditional Chinese medicine. So, *Artemisia* plants, especially *A. lavandulaefolia*, are suitable for being applied as trap plants for sustainable control of *A. lucorum* in tea plantations by means of supplying “push” stimuli.

## 5. Conclusions

*Apolygus lucorum* was the dominant mirid bug species in the tea plantation ecosystem, and a small amount of *Adelphocoris* spp. also occurred. *Apolygus lucorum* can infest a wide variety of crops including tea plants and caused serious yield losses of tender tea shoots during the spring growing season. In June and July, *A. lucorum* dispersed into tea plantations and seriously infested tea plants. *Apolygus lucorum* migrated from tea plants to and gathered on *H. scandens* and *Artemisia* plants at flowering stage in late August, and got nutrients from flowers for its survival, development, and fecundity. *Apolygus lucorum* reappeared on the flowering tea plants from the beginning of October and lay eggs in the dead parts of tea plants for overwintering. *Apolygus lucorum* densities on *H. scandens* and *A. lavandulaefolia* were relatively higher than those on *C. canadensis*, *A. annua*, and tea plants. Hence, the flowering *Artemisia* plants especially *A. lavandulaefolia* could be used as trap plants to attract and aggregate *A. lucorum*, and be more efficient to manage this tea pest in large-scale tea plantations. The use of *Artemisia* plants may also reduce the use of pesticides in tea crops and may contribute to a reduction of costs for the local tea farmers in the long run. In the future, this ecology control tactic may be incorporated as part of an integrated pest management (IPM) strategy for the control of *A. lucorum* and may be widely adopted by Chinese tea farmers.

## Figures and Tables

**Table 1:** Generalized linear models (GLM) parameters for effects of mirid bug species and tea-producing area on the numbers of mirid bugs.

Source	*df*	2016		2018	
		*F*	*p*-Values	*F*	*p*-Values
Mirid bug species	2, 63	45.267	**<0.001**	34.225	**<0.001**
Tea-producing area	2, 63	4.506	**0.015**	26.430	0.051
Mirid bug species × tea-producing area	4, 63	11.735	**<0.001**	41.279	**<0.001**

Bolded *p*-values indicate significant treatment effects (*p* < 0.05).

**Table 2:** GLM parameters for effects of host plant species and tea-producing area on the number of *A. lucorum*.

Source	*df*	2016		2018	
		*F*	*p*-Values	*F*	*p*-Values
Host plant species	4, 45	5.485	**0.001**	4.749	**0.002**
Tea-producing area	2, 45	0.035	0.965	2.383	0.102
Host plant species × tea-producing area	8, 45	6.190	**<0.001**	1.554	0.166

Bolded *p*-values indicate significant treatment effects (*p* < 0.05).

**Table 3:** Repeated measures MANOVA parameters for effects of host plants, sampling date, and interactions on population dynamics of *A. lucorum* on host plants.

Source	*df*	Experimental Site 1		Experimental Site 2	
		*F*	*p*-Values	*F*	*p*-Values
Host plant	4, 210	22.661	**<0.001**	29.259	**<0.001**
Sampling date	13, 210	29.679	**<0.001**	26.430	**<0.001**
Host plant × sampling date	52, 210	5.123	**<0.001**	12.836	**<0.001**

Bolded *p*-values indicate significant treatment effects (*p* < 0.05).
